# Role of glutamate in the development of visual pathways

**DOI:** 10.3389/fopht.2023.1147769

**Published:** 2023-03-03

**Authors:** Sriparna Majumdar

**Affiliations:** ^1^ Department of Psychology, Santa Clara University, Santa Clara, CA, United States; ^2^ Computer Science Department, City College of San Francisco, San Francisco, CA, United States

**Keywords:** retina, glutamate, development, synapse, extrinsic, neurogenesis, synapse formation

## Abstract

Glutamate is an important amino acid, metabolite and excitatory neurotransmitter, which is found in its free form in the extracellular spaces of the central nervous system (CNS). More than half of all synapses in CNS release glutamate. It is the main neurotransmitter driving the light responses in the retina. All types of photoreceptors, bipolar, ganglion and one type of glycinergic amacrine cells express specific subtypes of vesicular glutamate transporters and are the main source of endogenous glutamate in retina, besides Müller glia that are responsible for glutamate homeostasis, release and reuptake. Reduced or excessive extracellular glutamate was detected in the synaptic clefts of several naturally occurring or transgenic eye disease models, in which network rewiring and altered functions were observed. These led to the hypothesis that glutamate is one of the extrinsic signals for visual pathway development. This minireview examines experimental evidences supporting, or refuting, the influence of glutamate on prenatal and postnatal retinal development.

## Glutamate: A key metabolite and a neurotransmitter

L-glutamate is one of the 20 ubiquitous amino acids and the metabolic precursor of inhibitory neurotransmitter γ-amino butyric acid (GABA). Glutamate oxidation stimulates insulin secretion by pancreatic β-cells; it is an essential Kreb’s cycle metabolite, required to produce NADH inside mitochondria (1). Most importantly, glutamate is a neurotransmitter in the CNS, and serves as an extrinsic signal in neural development. Glutamate does not cross blood-brain barrier. Endogenous glutamate is produced from glutamine inside glutamatergic cells and released by vesicular glutamate transporters (VGLUT1-3) mediated exocytosis (2). Glial cells actively uptake extracellular glutamate using excitatory amino acid transporters (Glia specific EAAT1/GLAST, neuron specific EAAT2-5) (3). The glutamate concentration is 10,000–12,000 *μ*M/L inside neurons, but 0.5–2 *μ*M/L in the extracellular fluids. EAATs help to maintain this gradient. Glutamate activates its postsynaptic and extrasynaptic receptors upon release into the synaptic cleft (4). AMPA (GluA1-4), KA (GluK1-5), NMDA (GluN1, GluN2A-D) and Delta (GluD1-2) ionotropic glutamate receptors (iGluRs) vary in their sensitivity to agonists, antagonists, biophysical properties and distribution pattern. AMPA/KA iGluRs mediate fast excitatory neurotransmission, with characteristic rise time below 1 ms and deactivation kinetics varying widely depending on subunit composition. NMDA iGluRs are slower, require glycine as co-agonist and removal of Mg^2+^ block for activation. Metabotropic glutamate receptors (mGluR Groups I-III) modulate the excitability of neurons *via* excitatory G_s_/G_q_ (group I) or inhibitory G_i_/G_o_ (Group II-III) signaling pathways.

## Glutamate in retinal circuitry

**Figure 1:**
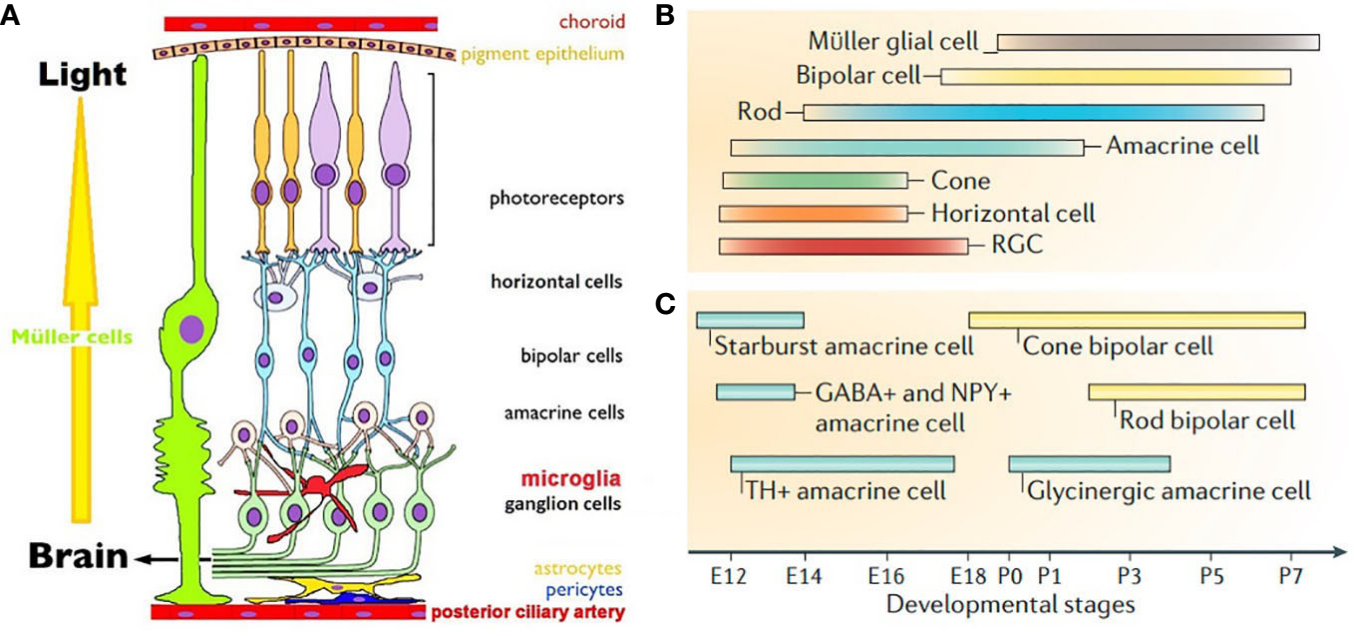
**(A)**. Schematic view of mammalian retina. Displaced RGCs, ACs and intra retinal blood vessels are not shown (7). **(B)**. Timeline of birth of retinal cells **(C)**. GABAergic ACs are born earlier than glycinergic ACs. Cone BPs are born before rod BPs. **(B, C)** are from Cepko, 2014 (8).

Vision is culturally and socially the most used, and hence most important of all animal senses (5). Retina is the first neural tissue in the visual pathway to sequester light, translate it into a change in membrane potential and transmit it to the synapses (6). There are three parallel image processing circuits in the retina that are driven by cones, with rods playing modulatory roles. They are object contours, color vision and motion detection circuits. Rods are monochromatic, 100 times more sensitive than polychromatic cones and drive night vision (**Figure 1A**) (9). These photoreceptors (PRs) form the outermost neural layer (ONL). Melanopsin containing intrinsically photosensitive retinal ganglion cells (mipRGCs) drive the blinking and pupillary light reflexes, photoentrainment of the circadian clock and encode the senses of ambient lighting condition (10). Glutamate drives PRs -> bipolars (BPs) -> RGCs neurotransmission. ON BPs express group III mGluR6 and horizontal cells (HCs) express iGluRs at the PRs ribbon synapses in the outer plexiform layer (OPL). OFF BPs form conventional synapses with PRs and express iGluRs. RGCs and ACs use iGluRs to receive glutamatergic inputs at ribbon dyads formed with BPs in the inner plexiform layer (IPL, 11). Light responses are fine-tuned by pre and postsynaptic mGluRs, mipRGCs and VGLUT3/glycine positive amacrine cells (ACs, 12–14). GABAergic HCs and GABAergic/glycinergic ACs reside in the inner nuclear layer (INL) and ganglion cell layer (GCL) and shape the temporal aspects of glutamatergic excitation. Gap junctions between retinal cells help in synchronizing the signals and lowering noise (15). RGCs relay the retinal output to lateral geniculate nucleus (LGN), suprachiasmatic nucleus (SCN), olivary pretectum (OP) and superior colliculus (SC). LGN relays visual information to striate cortex, SCN drives circadian rhythm, OP controls pupillary light reflex and SC controls eye movements required for focusing. Retina expresses VGLUTs in a complementary fashion. RGCs, Müller cell endfeets and some ACs and HCs express VGLUT2. PRs and BPs exclusively use VGLUT1, with the exception of a minority of cones that express VGLUT2 alongside VGLUT1 (16). VGLUT3 is expressed by a subtype of glycinergic ACs. Glutamatergic neurons in LGN, SCN, OP and SC express VGLUT2. Visual cortical areas use predominantly VGLUT1, except for VGLUT2 expressing layer 4 neurons, VGLUT3 expressing astrocytes and a sparse population of VGLUT3 neurons.

## Extracellular glutamate in eye diseases

At higher extracellular concentration glutamate is neurotoxic; excitotoxicity happens due to impairment in glutamate uptake by EAATs or blockade of metabolic pathways involving glutamate (17). Reduced synaptic glutamate, observed in retinitis pigmentosa and macular degeneration, is caused by mutations in one or multiple genes including iGluRs, mGluRs, exocytosis machinery, voltage gated and TRP channels, retinal pigment epithelium (RPE) and phototransduction related genes (18). Impaired glutamatergic neurotransmission impacts outer retinal lamination and initiates PR death. Moderate to dramatic changes are noticed in glutamate hyperexcitability conditions like ischemia, glaucoma, hyperglycemia induced diabetic retinopathy, migraine, schizophrenia, mood disorders, pathological pain, epilepsy, cerebellar ataxias, amyotrophic lateral sclerosis, Alzheimer’s and Huntington’s diseases (17, 19). In most of these diseases glutamate excitotoxicity initiates RGC death. In the retina of Alzheimer’s disease mice models, hyperexcitability was reported in early developmental stages, indicating early sign of pathogenesis.

## Development of the retina

Eye development in vertebrates follows a common theme across species. Optic vesicles of future eyes evaginate out bilaterally from diencephalon to form the optic cups. RPE and neural retina (NR) are generated from the outer and inner neural layers of optic cups. Cascades of mitotic cell division form clones of retinal progenitor cells (RPCs) competent to become a specific cell type at a given time during development. Early stages of development predominantly depend on extrinsic signals and switch to intrinsic during cell cycle exit (20). Intrinsic signals are typically transcription factors, protein inhibitors and microRNAs; extrinsic signals include neurotransmitters, growth factors and diffusible molecules (8, 20, 21). Extrinsic factors signal end of proliferation by transcriptional reduction of mitogens. There seems to be a predetermined probability of a constant percentage of RPCs to timely exit cell cycle at every phase of neurogenesis. Post-mitotic cells may become insensitive to the extrinsic signals as differentiation is guided by transcriptional auto-activation of cell specific markers. Differentiating cells secrete factors that prohibit more multipotent RPCs from exiting cell cycle, thereby controlling their own density. These processes ensure sequential birth of seven retinal cell types. RGCs are the first cells to be born, followed by HCs, cones, ACs, rods, BPs and Müller glia (**Figure 1B**) (8). Neurogenesis is followed by migration in the apico-basal space (apical: close to RPE, basal: close to posterior ciliary artery) to achieve correct mosaic and lamination pattern. Final stage of retinal development involves synapse formation and pruning, until each cell type achieves adult like morphology, stratification pattern and functional synapses. Synaptic partners are brought together by cell adhesion molecules and extracellular matrix proteins. Neurons with no functional synapses undergo programmed cell death.

## Glutamate in prenatal retinal development

Earliest works on the role of glutamate in retinal development concentrated on detecting glutamate in developing retina and evaluating effects of glutamate agonists and antagonists (22). Extracellular glutamate concentration is very high in prenatal retina, dropping abruptly at birth. Exogenous kainate acts as a trophic factor at low concentration and promotes HCs neurite sprouting into INL; higher concentration causes ablation of HCs in adult and developing rodent retina (23, 24). At P1, exogenous KCl causes nearly 2-fold increase in extracellular glutamate. This is even before glutamatergic synapses formed in OPL (P2-P5) and IPL (P6-P8) suggesting presence of extrasynaptic glutamate release mechanisms in the embryonic retina (25). VGLUT2 is detected in CNS beyond E9.5 and is critically required for prenatal development. Embryonic VGLUT1 transcripts were detected nowhere except olfactory cortex (26). The developmental role of PRs specific splice variant VGLUT1v is yet to be determined (27). Although VGLUT3 expression is limited to a subset of ACs in the retina, it is the primary VGLUT in inner ear. VGLUT3 expression is seen at E18 and beyond in CNS. Whether VGLUT2 or another VGLUT transiently expresses in embryonic RPCs and their expression switches at birth, similar to developmental switch from VGLUT2/VGLUT3 to VGLUT1 in the pyramidal cells of cerebellum, is not known.

**Figure 2:**
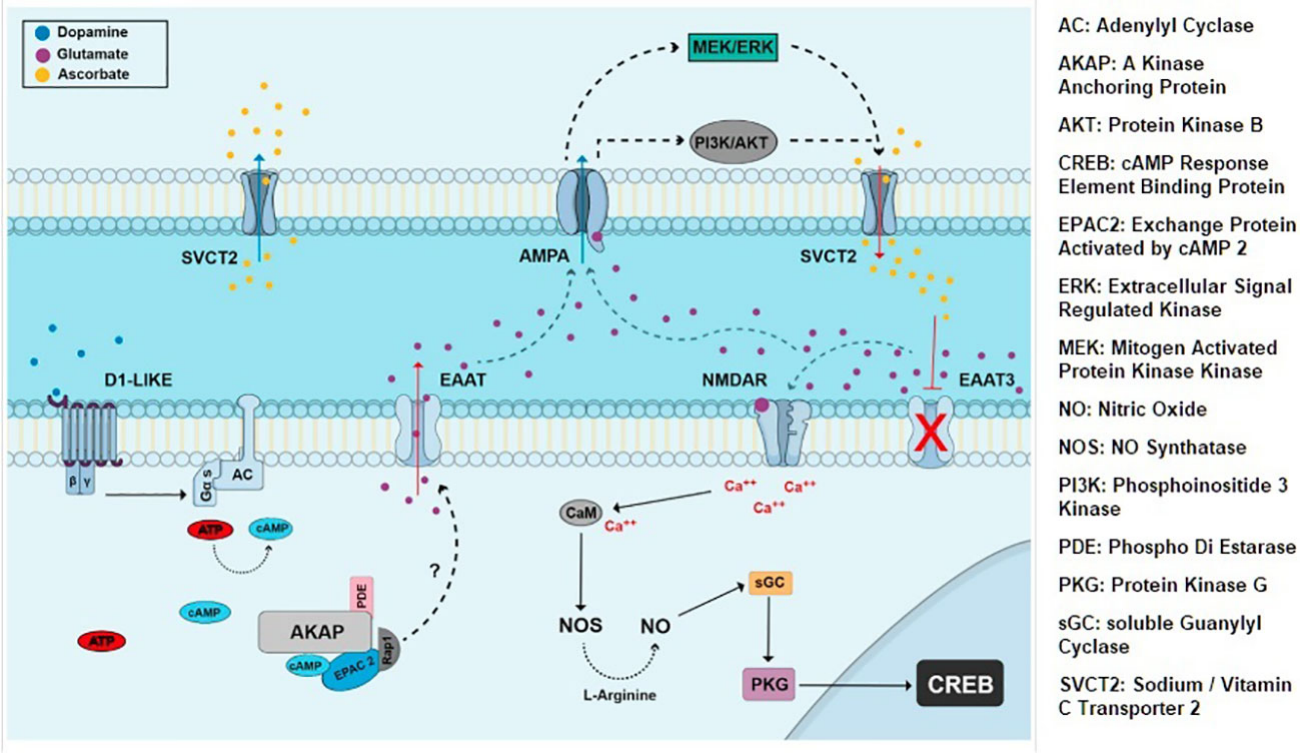
Extrinsic signaling involving dopamine, glutamate, EAATs and NMDA iGluRs. CREB is activated by NMDA and NO dependent cGMP/PKG pathway and affects neuronal proliferation and survival choices in chick retina (29).

iGluRs and mGluRs express at E14.5 and beyond; neuron-specific EAAT2 and EAAT3 express E14 onwards (28). Embryonic AMPA/KA receptors mediate exit from cell cycle by activating cyclin dependent kinase2 (cdk) inhibitor pathway; in contrast, mGluRs support proliferation. NMDA receptors mediate the neurotoxic effects of glutamate in visual pathways and cerebellum. In the developing chick retina NMDA receptors mediate choice between neuroprotection and programmed cell death by switching phosphorylation state of CREB protein (**Figure 2**) (29). In ischemia EAATs dependent release of glutamate into the extracellular space causes NMDA receptor mediated apoptosis. Taken together, EAAT2-3, iGluRs and mGluRs drive prenatal glutamatergic extrinsic signaling that influence choice between proliferation vs. differentiation and cell survival vs. programmed cell death.

RGCs axons project to LGN, SCN, OP and SC prenatally. Glutamate’s role in RGC axon guidance is not yet understood. A recent study reports that postsynaptic axon guidance effector abl2 kinase reduces iGluR currents. Abl2 knockdown causes dynamic reduction in the number of distal excitatory synapses in hippocampal cell cultures (30). Similar study has not been done in the retina. Number of axon projections to SC is altered in acetylcholine receptor beta2 knockout, presumably by tip-over glutamatergic excitation (31). This claim needs to be further validated.

## Glutamate in postnatal retinal development

Retinal morphogenesis and lamination starts prenatally and continues postnatally, followed by synaptogenesis and network maturation that continues even after eye opening in rodents. At birth cones, some rods, HCs, RGCs and GABAergic ACs are already undergoing differentiation (**Figures 1B, C**). At P0 neuroblast layers have some rudimentary laminar structure; by P14 adult like laminar organization becomes apparent (21). Reese and colleagues showed that cones, HCs, ACs and RGCs may migrate tangentially, in addition to radially during mosaic formation (32). Rods, BPs and Müller glia are born from a single RPC that creates clonal radial columns in the retina, requiring postnatally born cells to migrate minimally. Migration effector protein reelin is expressed in RGCs, HCs, OPL and IPL and enhances Ca^2+^ conductance of NMDA receptors. In reeler mice rod BPs density is reduced, presumably due to altered NMDA receptor mediated choice of cell survival vs. apoptosis (33). Precise role of extracellular glutamate as an attractant or repellent in retinal migration is yet to be elucidated.

It is debated whether synapses form first and axo-dendritic arbors of pre and postsynaptic partners move together to find their desired strata. Paracrine glutamatergic signaling facilitates synapse formation by acting onto GluN2B extrasynaptic NMDA receptors in developing retina (34). Formation of ON pathway is delayed in dark reared retina, or in absence of glutamatergic input from mipRGCs (35). Synaptic targeting of ON BPs and ON RGCs dendrites does not depend on glutamate release from PRs and BPs, but fewer synapses form in reduced glutamate conditions (36). Thus, excitatory synapse formation in ON pathway requires glutamatergic signal. Role of glutamate in OFF pathway formation remains inconclusive.

Waves of transient, retina-wide correlated bursts of spiking are seen in developing GCL. Gap junctions mediate these waves in E17-P1 (stage I), acetylcholine in P2-P9 (stage II) and glutamate in P10-P14 (stage III) rodent retinas. Glutamatergic waves are not detected in VGLUT1 knockout (37). Ablating VGLUT2 expressing mipRGCs causes incomplete segregation of ipsi and contralateral RGC projections in LGN (38). Precocious glutamatergic wave causes purely ON or OFF projections in SC, whereas ON and OFF projections overtly segregate in LGN. RGCs dendrites diffusely stratify at P1. Segregation of their dendrites into ON and OFF sublamina of IPL requires cessation of group III mGluR signaling, as exogenous application of selective agonist APB severely delays this process (39). These indicate glutamate’s role in RGCs network maturation. Recent evidences suggest that mipRGCs influence VEGF and oxytocin dependent vasculature and synapse formation, implying bigger impact of light mediated glutamatergic signaling on brain and behavior (40).

## Conclusion

Retinal cells are extremely diverse in their morphology and function. To this date 1 rod, 2 or more cones, 1 rod BP, 3 HCs, 1 Müller glia, 9 cone BPs, 30-60 ACs, 3-5 mipRGCs and 20-30 RGCs have been described. Establishing a multifunctional network containing millions of neurons in a 0.2 mm thick retina requires timed interplay between extrinsic, intrinsic and trophic signals. Extrinsic glutamatergic signaling is crucial for developmental choice between proliferation and cell cycle exit, as well as survival and apoptosis; in postnatal retina it affects synapse formation and network maturation. Role of glutamate in neural migration and axon guidance hasn’t been clearly identified.

## Author contributions

SM reviewed the relevant articles, conceptualized the review and wrote the manuscript. The author confirms being the sole contributor of this work and has approved it for publication.
